# The gap effect reduces both manual and saccadic inhibition of return (IOR)

**DOI:** 10.1007/s00221-019-05537-8

**Published:** 2019-04-05

**Authors:** Łukasz Michalczyk, Jacek Bielas

**Affiliations:** Institute of Psychology, Jesuit University Ignatianum in Krakow, Kopernika 26, 31-501 Krakow, Poland

**Keywords:** Inhibition of return, Eye movements, Visual attention, Selection-for-action, Selection-for-perception

## Abstract

Inhibition of return (IOR) is the effect of slower responses to validly than invalidly cued targets. The discovery of IOR raised controversy as to whether it has two “flavors”, i.e., attentional/perceptual and motoric, or whether it is a homogeneous visual-motor phenomenon that should be understood in terms of the preparation of different effectors (mainly eye movement). Since manipulation of fixation offset (0 and 200 ms gap) is believed to affect the latency of saccades, we measured its influence on saccadic and manual IOR with a simple keypress response when eye movements were forbidden. In the two experiments which we carried out, the fixation offset decreased IOR in both the saccadic and the manual conditions. The results suggest the limitations of the attentional hypothesis, which assumes that manual IOR is independent of the motoric component; they are also in line with the tenets of the oculomotor hypothesis of IOR.

## Introduction

When the time interval (SOA) between a pair of stimuli is longer than approximately 300 ms, reaction times to targets presented at previously stimulated locations are longer than to targets presented at new locations. This effect is referred to as inhibition of return (IOR) and is understood as a process of attentional selection that is measured in cuing tasks by a response to an on-screen target that was preceded by a cue. When a cue and a target occupy the same location, the response is generally slower than when they are presented in separate locations. Depending on the type of response, manual and saccadic IOR are distinguished. The saccadic condition demands a saccade to the target location (Posner et al. 1985; Klein and MacInnes 1999); the manual condition demands a keypress response when the target appears on the screen, but the eyes have to be fixated on the fixation point (Berlucchi et al. 1981; Posner and Cohen 1984).

According to the Premotor Theory of Attention (PToA, Rizzolatti et al. 1987, 1994), the same systems which control the preparation of goal-directed actions also control sensory selection. Therefore, saccadic IOR and manual IOR should be processed by the same sensory-motor mechanism. In the case of visual selection, the preparation of eye movement has a dominant role. A significant body of research points to the close link between manual and saccadic IOR and emphasizes the crucial role of the oculomotor system (e.g., Christie et al. 2013; Galfano et al. 2004; Maylor 1985; McGowan et al. 1998; Posner et al. 1985; Rafal et al. 1989; Michalczyk et al. 2018, but, for a contrary view, see also Smith et al. 2012).

However, a considerable body of research also shows dissociation between saccadic and manual IOR (Hilchey et al. 2014, 2016; Hunt and Kingstone 2003; Kingstone and Pratt 1999; Taylor and Klein 2000; Zhang and Zhang 2011) and, thus, supports another approach to manual IOR, i.e., as an attentional phenomenon which is independent of oculomotor activity. According to the main assumption of attention-based IOR, this phenomenon should appear in the manual condition, because, in this variant of the cuing task, the manual response is not spatially directed to the stimuli and eye movements are also actively inhibited (e.g., Hilchey et al. 2016; Hunt and Kingstone 2003; Ivanoff et al. 2002; Taylor and Klein 2000). In consequence, according to this approach, two distinct mechanisms of IOR exist: manual/attention IOR and saccadic/motor IOR.

One area that can be used to study oculomotor and attentional views on IOR is the gap paradigm. There are two main variants of the gap effect task: a 200 ms gap (“true gap”), for which the fixation point is removed 200 ms before target onset (Saslow 1967), and a 0 ms gap (fixation offset effect; FOE), for which it is removed at the moment of target presentation (Kingstone and Klein 1993; Fendrich et al. 1999). Typically, this manipulation results in faster saccadic movements to the target compared with the condition in which the fixation point remains on the screen throughout the whole trial. Two components are usually considered to cause the gap effect. The first is related to the activity of the fixation system, i.e., competition between the opposing processes of fixation reflex and visual grasp reflex (e.g., Dorris and Munoz 1995; Findlay and Walker 1999; Kingstone and Klein 1993); the second is associated with warning signals and is related to the state of readiness of the oculomotor system (Model of Saccade Generation; Findlay and Walker 1999) or the saccade program, which is partially prepared before target onset (the Motor Preparation Hypothesis; Pare and Munoz 1996).

Many studies have implicated that the *superior colliculus* (SC) is crucial in the programming of eye movement (Schiller 1977), but it is also important in generating gap and IOR effects. For instance, both IOR and gap effects are present at birth (e.g., Simion et al. 1995; Farroni et al. 1999). In newborns, the gap effect does not appear with stimuli that do not activate the SC (Farroni et al. 1999) and saccadic IOR is greater in the temporal than in the nasal visual field (Simion et al. 1995). This temporal–nasal asymmetry has also been found in both saccadic and manual IOR in adults (Rafal et al. 1989, 1991), and is probably associated with a characteristic of the SC, which, via retinotectal pathways, achieves more inputs from temporal than nasal parts of the visual field (Farroni et al. 1999; Rafal et al. 1991; Simion et al. 1995). Given that the gap effect and IOR depend on eye programming, one might infer that they should interact with each other.

However, according to attention-based IOR, the gap effect, as caused by motor components, should not interact with manual IOR. Indeed, Hunt and Kingstone (2003) found that the 0 ms gap affected only saccadic IOR, which decreased compared to the condition in which the fixation stimulus remained visible. Hunt and Kingstone’s study is probably the only research which has attempted to distinguish saccadic and manual IOR using the gap paradigm (e.g., Klein and Hilchey 2011). This pattern of results has not been observed in studies in which the 200 ms gap paradigm was used. Souto and Kerzel (2009) did not show any interaction between the 200 ms gap effect and IOR in both manual and saccade conditions. Moreover, Abrams and Dobkin (1994), also in a 200 ms gap paradigm, demonstrated the opposite to Hunt and Kingstone’s (2003) pattern of saccadic IOR: the disappearance of the fixation point increased IOR. This result was later replicated by Guimaraes-Silva et al. (2004). According to Souto and Kerzel (2009), in the case of saccadic IOR, these discrepancies in results suggest that both types of gap could engage different processes. However, there is no obvious reason that the gap effect should affect saccadic IOR differently in the 0 ms gap and in the 200 ms gap conditions. On the contrary, as a warning signal improves readiness to react (Findlay and Walker 1999) and influences the preparation of movement (Pare and Munoz 1996), it could be expected that, in a 200 ms gap condition, not the direction would change, but rather the effect size would increase compared to the 0 ms gap condition.

The lack of impact of the gap on manual IOR in both the 0 ms gap (Hunt and Kingstone 2003) and the 200 ms gap condition (Souto and Kerzel 2009) is inconsistent with research that indicates a close link between manual IOR and preparation of eye movement. Although these results support the attention-based IOR hypothesis, the meaningful interpretation of such a negative result requires greater statistical power. Thus, this result could also be considered as a case of a type two statistical error, which, therefore, encourages a reexamination of the relation between the gap effect and manual IOR (Cohen 1977). There is also a possibility that the missed interaction between the gap and manual IOR may be caused by procedural reasons related to the type of response required. In both previous studies, manual IOR was measured by an uncrossed choice keypress response; how the participants were supposed to respond was not specified (with one or with both hands). However, it may be assumed that, in Hunt and Kingstone’s study, the participants used both hands to respond, e.g., the left hand pressed the left key (the Z key on keyboard) and the right hand pressed the right key (the slash key). In Souto and Kerzel’s study, this is harder to determine, because the participants had to press the left key or the right key on a gamepad, so this could also have been a unimanual choice task. In general, a choice response is more difficult to perform than a simple keypress, because it requires a more complex reaction (Bekkering et al. 1996). For instance, choosing between two possible responses (e.g., left key or right key) requires activation of the correct reaction and inhibition of others, and it is always possible to make an error by executing two responses at the same time. In contrast, simple keypress responses (similarly to eye movements) are mutually exclusive: choosing one movement excludes the ability to perform another. It is also known that, in an uncrossed choice keypress response, the right hand is faster than the left hand (Berlucchi et al. 1971; Wallace 1971; Brebner et al. 1972; Anzola et al. 1977), but this spatial compatibility is not apparent in a simple reaction time (Anzola et al. 1977; Berlucchi et al. 1971). Anzola et al. (1977) argue that this effect of the superiority of the right hand could not be explained purely by the anatomical fact that the dominant hand reacts faster. According to their hypothesis, the need to decide which hand has to be moved could activate the left motor (or premotor) hemisphere, which would favor a response of the right hand. Based on this, it could be assumed that the planning and execution of movements in a choice keypress response, e.g., when both hands are engaged, could influence the results and potentially disrupt the gap and manual IOR interaction.

It is known that the IOR effect occurs not only in a choice response but also in a simple reaction time task, in which it is also considered to be triggered by attention processes (Posner and Cohen 1984; for review: Wright and Ward 2008). Therefore, we decided to reexamine the relation between the gap effect and manual IOR, but with the use of a simple keypress response instead of a choice response.

The primary aim of this study was to challenge a key prediction of attention-based IOR, according to which IOR that is generated independently of the motor system should appear in a manual response when moving the eyes is forbidden (e.g., Taylor and Klein 2000; Hilchey et al. 2016). If this is correct, the gap effect should only impact saccadic IOR, whereas the interaction between the gap effect and manual IOR would be in line with alternative oculomotor priming hypotheses (Rafal et al. 1989; Rizzolatti et al. 1987, 1994). We carried out two experiments to address this question: in the first, we used the 0 ms gap, and in the second, we used the 200 ms gap paradigm.

## Experiment 1

### Methods

#### Participants

Twenty-eight university students (25 female, 3 male; aged 19–24)^1^ participated in the present study for course credits. They were all right-handed. The participants were unaware of the purpose of the experiment. All had normal or corrected-to-normal vision. Each of them took part in two sessions on 2 separate days. This experiment was approved by The Research Ethics Committee at the Jesuit University Ignatianum in Krakow and carried out in accordance with the Code of Ethics of the World Medical Association (Declaration of Helsinki). Individuals gave informed consent prior to their participation in the study.

#### Apparatus and stimulus

Participants were seated 57 cm away from a 17 CRT monitor (85 Hz refresh rate) with their heads stabilized in a chin and forehead rest. Eye movements were recorded monocularly by an EyeLink1000 plus system sampling at 500 Hz.

All stimuli were presented on a gray background. Two black open squares (2° × 2°, edge thickness: 0.1°) acting as placeholders were displayed 7° to the left and right of the black fixation dot (0.2°). The cue was a black frame (0.3°) that was formed by thickening the edges of a placeholder. The target was a black dot (0.8°) presented centrally within a placeholder for 1000 ms (until response).

#### Procedure

**Fig. 1:**
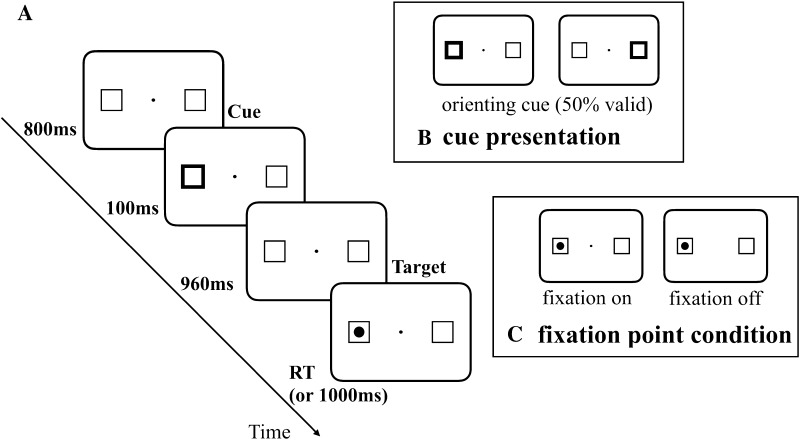
Experimental procedure: **a** an example of the event sequence in the cuing task, **b** the cue presentation, and **c** an example of fixation point condition

Each trial started with an automatic drift correction procedure; when it failed, the subject had to be recalibrated. Given accurate calibration and drift correction, the red dot changed color to a black fixation point, which, at the same time, started the trial sequence, as shown in Fig. 1.

A fixation point and two placeholders were presented for 800 ms, and then, a cue appeared for 100 ms, randomly to the left or right of the fixation point. One thousand and sixty milliseconds after the onset of the cue (SOA), the target was presented; the subjects had to react to it by a manual keypress or by executing a saccade towards the target as quickly as possible. The fixation point could either remain on the screen or disappear simultaneously with the onset of the target. The target was present on the screen until the answer was recorded (or it disappeared after 1000 ms). The duration of the inter-trial interval (ITI) was at least 2000 ms, plus the variable time, it took each subject to complete the automatic drift correction (from 0 to 5000 ms).

Each subject participated in two sessions (on 2 different days) and performed the same task but with varying constraints of response. The order of sessions was balanced across subjects.

In the manual condition, participants were instructed to keep their eyes on the fixation location during the trial (even when the fixation point disappeared) and press using right hand the spacebar as quickly as possible in response to the target. The reaction time was measured as latency from the onset of the target until the registration of the button press. In the saccadic condition, participants were instructed to keep their eyes on the fixation point and make a quick saccade to the target after its onset. The saccadic reaction time (SRT) was defined as the latency of saccades that landed within a 2° boundary region surrounding the target. The algorithm for detecting saccades determined an eye movement to be a saccade when a fixed velocity threshold of 30°/s and an acceleration threshold of 8000°/s^2^ were exceeded.

If a subject broke fixation or responded before the onset of the target, or a successful saccade/manual response was not made within 1000 ms of the target onset, on-screen error feedback was given to the participant and the trial was placed in the pool of unfinished trials to be completed later. Each session consisted of four blocks of 32 trials each (a total of 128 trials per session), preceded by 48 training trials. In each block, there were 16 (50%) valid trials in which targets were presented at cued locations, and 16 (50%) invalid trials in which the targets were displayed at locations opposite the cue. In each block, the fixation offset variable was randomized and distributed equally between valid and invalid trials. The offset manipulation had two levels: the fixation dot could either remain on the screen (fixation on) or disappear from the display simultaneously with the onset of the target (0-ms gap).

### Results

Repeating error trials (fixation break and misses) at the end of every block lengthened the manual cuing task on average by 34 trials (26%) and the saccade task on average by 28 trials (21%). In addition, in manual and saccadic RT data, lower bounds of 100 ms (0.2% trials) and 80 ms (1%) were, respectively, set to exclude anticipatory responses. The number of repetitions that a subject had to make did not correlate significantly with RTs or SRTs in any condition. For every subject and experimental cell, the remaining trials were submitted to a trimming procedure with a cut-off criterion of 3 SD using the “prepdat” R Package (Allon and Luria 2016). As a result, 22 trials (0.5%) were additionally removed in the manual task, and 3 trials were removed in the saccadic task (0.08%).

We calculated median response times for each subject; these were then submitted to two separate, repeated-measure ANOVAs, one for the saccadic and one for the manual responses. In both analyses, the fixation offset (fixation on and 0 ms gap) and validity (cued–uncued) were the within-subject factors. Sensitivity power analysis by use of G-Power 3.1.9.3 showed that our sample size allows detecting the minimal detectable effect (MDE) of Cohen *d* = 0.48 (power = 0.8; *α* = 0.05).

#### Saccade RT (SRT)

**Fig. 2:**
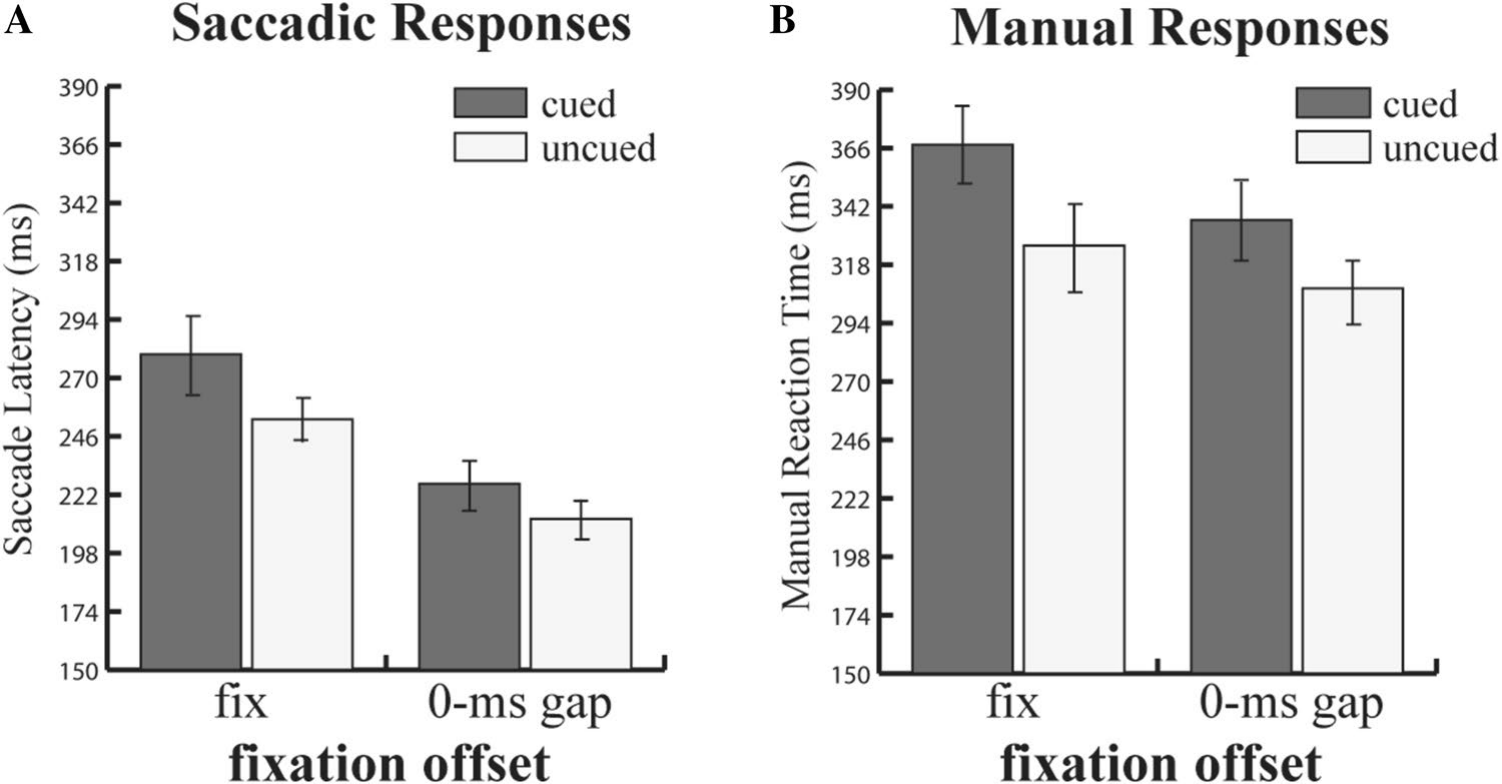
**a** Mean saccade latencies and **b** manual reaction times (in ms) as a function of the fixation offset and cuing condition in experiment 1. The fixation offset and IOR interaction is statistically significant for both the saccadic response (shown in the left panel) and manual response (shown in the right panel). Error bars show ± SEM

The SRT data are shown in Fig. 2a. There was a main effect of fixation offset manipulation, with saccades being faster when the fixation point was removed (219 ms, SD = 7.12) compared to when it remained on the screen (266 ms; SD = 9.22) [*F*(1,27) = 126.1; *p* < 0.0001; $\eta_{p}^{2}$ = 0.82]. There was also a reliable validity effect: latencies of saccades were longer to the previously cued locations (253 ms; SD = 8.2) as compared to uncued locations (232 ms; SD = 7.56) [*F*(1,27) = 68.7; *p* < 0.0001; $\eta_{p}^{2}$ = 0.72]. Importantly, the effects of the two factors interacted [*F*(1,27) = 11.41; *p* < 0.01; $\eta_{p}^{2}$ = 0.30]: the inhibition of return (uncued–cued SRT) was larger for the fixation on condition (IOR 27 ms, Cohen *d* = 0.74) than the 0 ms gap (IOR 14 ms, Cohen *d* = 0.51) condition (*M*_diff_ = 13 ms, Cohen *d* = 0.77).

#### Manual RT

Figure 2b also shows the results for manual responses. Two main effects, fixation offset [*F*(1, 27) = 65.7; *p* < 0.001; $\eta_{p}^{2}$ = 0.71] and validity [*F*(1,27) = 55.9; *p* < 0.001; $\eta_{p}^{2}$ = 0.67] were significant. RTs in the 0-ms gap were faster (322 ms; SD = 11.04) than in the fixation on condition (346 ms; SD = 9.36). RTs to validly cued trials were slower (351 ms; SD = 10.21) than to invalidly cued trials (317 ms; SD = 10.86). The interaction between these two factors was also significant [*F*(1,27) = 8.93; *p* < 0.01; $\eta_{p}^{2}$ = 0.24]. The inhibition of return (uncued–cued RT) was larger for the fixation on condition (IOR 42 ms, Cohen *d* = 0.94) than for the 0 ms gap (IOR 28 ms, Cohen *d* = 0.76) condition (*M*_diff_ = 14 ms, Cohen *d* = 0.49).

### Discussion

The objective of the present experiment was to investigate the influence of the gap effect (0-ms gap) on inhibition of return. Our results show that the IOR effect in both manual and saccadic responses was smaller when the fixation point was removed than when it remained present until the end of the trial. The presence of an interaction between the 0 ms gap and saccadic IOR replicated Hunt and Kingstone’s (2003) results; therefore, it supported the hypothesis of a direct link between saccadic IOR and preparation of eye movement. On the other hand, we also found an interaction between the 0 ms gap effect and manual IOR.

Discrepancies between our results and Hunt and Kingstone’s may be due to three differences. First, as Souto and Kerzel (2009) noticed, Hunt and Kingstone probably did not perform any data trimming, so outlying data points could have contaminated their results. This may explain why a gap effect in the choice keypress response condition was missed, although a small (~ 6–9 ms) but significant effect is usually observed in similar studies (Gómez et al. 1998; Bekkering, et al. 1996; Souto and Kerzel 2009). Second, in contrast to Hunt and Kingstone, in the manual task, we used the simple instead of the choice keypress response and obtained a relatively higher gap effect (24 ms). This may indicate that the simple detection response is more sensitive to the gap effect than the choice keypress response. Third, compared with their study, other changes in the procedure (such as change of brightness from bright to dim stimuli, feedback from fixation breaks or missed reactions, and the need to repeat these trials) might have further improved the statistical power in our study.

All in all, we found that the gap effect—at least in a 0 ms gap variant of the task—interacts with both manual and saccadic IORs. This suggests that not only saccadic IOR but also manual IOR could share common mechanisms with the preparation and programming of eye movements.

## Experiment 2

The decrease of saccadic IOR and manual IOR due to the 0 ms gap contradicts the results obtained in similar studies in which the “pure” gap effect (200 ms gap) was applied (Abrams and Dobkin 1994; Guimaraes-Silva et al. 2004; Hunt and Kingstone 2003; Souto and Kerzel 2009). Therefore, we conducted a second experiment in which we used the 200 ms instead of the 0 ms gap paradigm. However, to improve statistical power, we left the essential parts of the procedure from experiment 1 unchanged, in particular the type of manual reaction and stimulus.

### Methods

#### Participants

Twenty university students (14 female, 6 male; aged 19–24) participated in the present study for course credits. They were all right-handed. The participants were unaware as to the purpose of the experiment. All had normal or corrected-to-normal vision. Each of them took part in two sessions on separate days. Experiment was approved by The Research Ethics Committee at the Jesuit University Ignatianum in Krakow and carried out in accordance with the Code of Ethics of the World Medical Association (Declaration of Helsinki). Individuals gave informed consent prior to their participation in the study.

#### Apparatus, stimulus, and procedure

All aspects of this experiment were the same as in experiment 1, with the exception that the fixation dot could either remain on the screen (fixation on) or disappear from the display 200 ms before the onset of the target (200-ms gap). As in Experiment 1, the subjects participated in two sessions. In the first, they had to make a saccade to the target and in the second a manual response. As in the previous experiment, in each session, they completed four blocks of 32 trials each (a total of 128 trials per session), preceded by 48 training trials.

### Results

Repeating error trials (fixation break and misses) at the end of every block lengthened the manual cuing task on average by 19 trials (15%), and the saccade task on average by 20 trials (16%). In addition, to exclude anticipatory responses, in manual RT data, a lower bound of 100 ms (3% trials) was set, while, in saccadic RT data, a lower bound of 80 ms (0.2%) was set. The number of repetitions that a subject had to make did not correlate significantly with RTs/SRTs in any condition. For each participant and experimental cell, the remaining trials were submitted to a trimming procedure with a cut-off criterion of 3 SD using the “prepdat” R Package (Allon and Luria 2016). As a result, 75 trials (3%) were additionally removed in the manual task and 28 trials in the saccadic task (1%).

We calculated median response time for each subject. The medians were then submitted to two separate, repeated-measures ANOVAs, one for the saccadic and one for the manual responses. In both analyses, the fixation offset (fixation on and 200-ms gap) and validity (cued–uncued) were the within-subject factors. The power analysis of sensitivity shows that, with a criterion of the power 0.80, the simple size of 20, and *α* = 0.05, the MDE should achieve at least a value of Cohen *d* = 0.57.

#### Saccade RT (SRT)

**Fig. 3:**
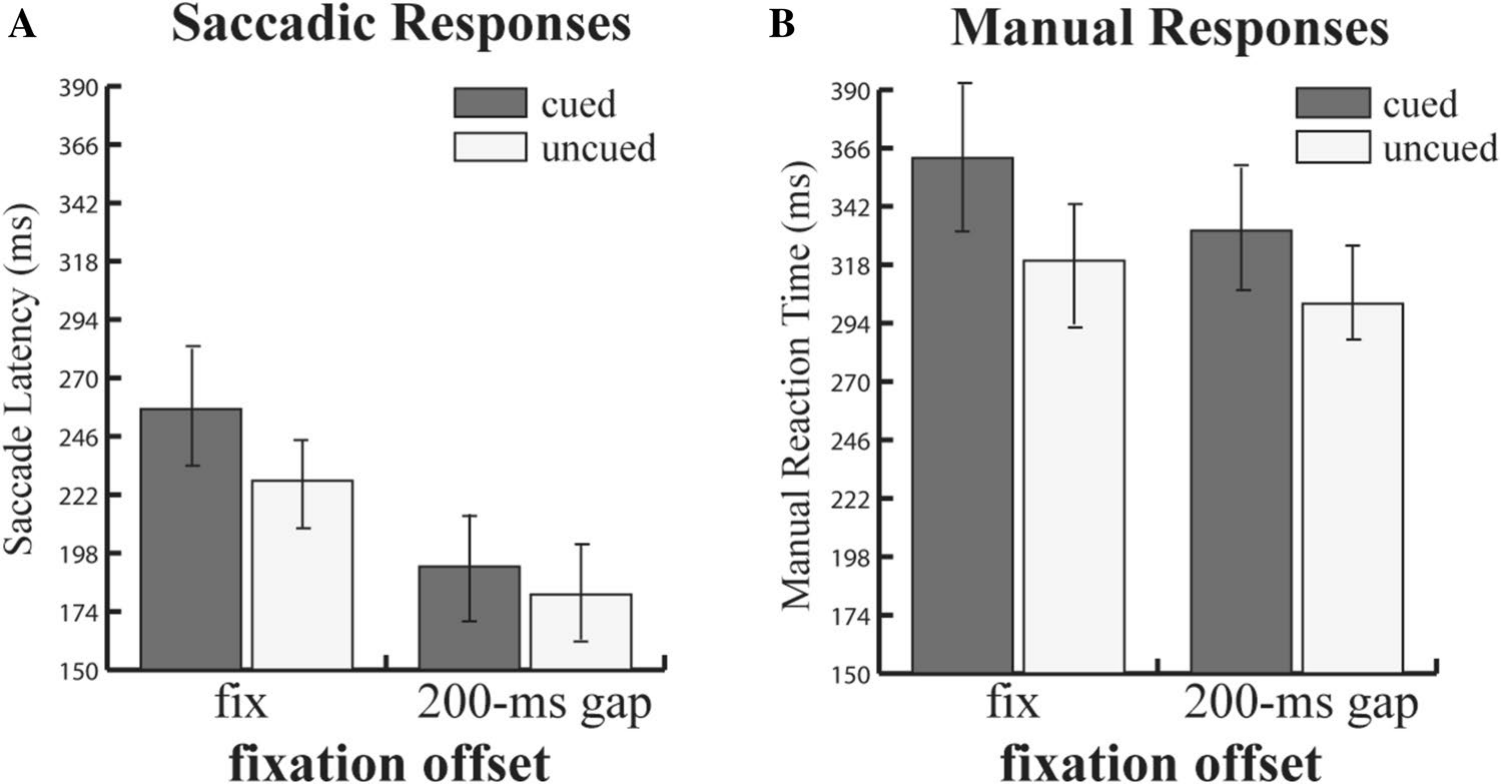
**a** Mean saccade latencies and **b** manual reaction times (in ms) as a function of gap and cue manipulation in experiment 2. Error bars show ± SEM

The SRT data are shown in Fig. 3a. There was a main effect of fixation offset manipulation, with saccades being faster when the fixation point was removed (186 ms; SD = 13.89) compared to when it remained on the screen (242 ms, SD = 14.55) [*F*(1,19) = 37.91; *p* < 0.001; $\eta_{p}^{2}$ = 0.66]. There was also a reliable validity effect: latencies of saccades were longer to the previously cued locations (224 ms; SD = 11.54) as compared to uncued locations (203 ms; SD = 14.22) [*F*(1,19) = 28.2; *p* < 0.001; $\eta_{p}^{2}$  = 0.58]. The effects of the two factors interacted [*F*(1, 19) = 32.9; *p* < 0.001; $\eta_{p}^{2}$  = 0.63]; the inhibition of return (uncued–cued SRT) was larger for the fixation on condition (IOR 31 ms, Cohen *d* = 0.66) than for the 200-ms gap (IOR 11 ms, Cohen *d* = 0.24) condition (*M*_diff_ = 20 ms, Cohen *d* = 1.04).

#### Manual RT

Figure 3b shows the results for the manual RTs. There was a significant main effect of fixation offset [*F*(1,19) = 21.34; *p* < 0.001; $\eta_{p}^{2}$ = 0.52] and validity [*F*(1,19) = 64.55; *p* < 0.001; $\eta_{p}^{2}$ = 0.77]. RTs in the 200 ms gap condition were faster (318 ms; SD = 18.43) than in the fixation condition (342 ms; SD = 18.19). RTs to validly cued trials were slower (349 ms; SD = 19.55) than in invalidly cued trials (311 ms; SD = 16.90). The interaction between these two factors was also significant [*F*(1,19) = 5.4; *p* < 0.05; $\eta_{p}^{2}$  = 0.22]. Inhibition of the return effect was larger in the fixation on condition (IOR 46 ms, Cohen *d* = 0.76) as compared to the 200 ms gap (IOR 31 ms, Cohen *d* = 0.58) condition (*M*_diff_ = 15 ms; Cohen *d* = 0.56).

### Discussion

As in experiment 1, in which we used a different gap paradigm (0 ms gap), we observed again that the gap effect (200 ms gap) interacts with saccadic and manual IOR. Consequently, this result supported the oculomotor hypothesis of manual IOR. Moreover, in both IORs (saccadic and manual), the direction of interaction was the same as in experiment 1: the magnitude of the IOR effect decreased when the fixation point disappeared 200 ms before the target onset.

Our results contrast with those of Abrams and Dobkin (1994) and Guimaraes-Silva et al. (2004), both of whom showed an increase of saccadic IOR after the 200 ms gap. The cause of this discrepancy is unclear and worthy of further research, but we speculate that it may have arisen mainly for procedural reasons. We conducted a more typical IOR procedure than that of Abrams and Dobkin and Guimaraes-Silva et al. Abrams and Dobkin’s results are considered unusual and are, therefore, probably responsible for the surprising lack of IOR in the fixation condition, in which ordinarily a reliable IOR effect is observed (Hunt and Kingstone 2003; Klein and Hilchey 2011). In the study of Guimaraes-Silva et al. (2004), the same stimulus (green light-emitting diodes) had different functions (i.e., cue, target, and fixation point). Use of this kind of stimuli set has been criticized as it can cause some perceptual confusion and, in consequence, impact the participants’ response strategy (Klein and Hilchey 2011). Moreover, in contrast to our study, Guimaraes-Silva et al. (2004) blocked the fixation offset condition, which could have affected the results by increasing the significance of the warning signal.

## General discussion

Our study aimed to determine how the disappearance of the fixation point would influence the magnitude of saccadic and manual IOR. We conducted two experiments that slightly differed procedurally: in the first, we examined the 0 ms gap, and in the second, we examined the 200 ms gap. We found that, independently of the task variant, the gap effect interacts with both saccadic and manual IOR. The following results will be discussed in turn:

1. The 0/200 ms gap interaction with saccadic/manual IOR.
2. The possible neuronal mechanism of IOR.
3. The interpretative limitations of obtained results.

### The 0/200 ms gap interacting with saccadic/manual IOR

In experiment 1, we showed that the 0 ms gap affects saccadic IOR; this is consistent with the previous studies (Hunt and Kingstone 2003; Souto and Kerzel 2009). In experiment 2, we showed that the 200 ms gap also affects saccadic IOR in the same direction as the 0 ms gap and—contrary to some previous predictions (Abrams and Dobkin 1994; Guimaraes-Silva et al. 2004; Souto and Kerzel 2009)—this suggests that both share a common mechanism.

The gap facilitates eye movement by influencing saccadic preparation (e.g., Fendrich et al. 1999; Rolfs and Vitu 2007), so its interaction with saccadic IOR seems a natural consequence. However, a coupling between the gap and manual IOR is not apparent, because the manual response is not directed to the targeted location, and therefore, any goal-directed preparation of the eyes is not necessary. Taylor and Klein (2000) assume that, in cases of cuing tasks in which the eyes must be fixated, manual IOR is generated by attentional processes that are distinct from eye movement preparation (see also: Hilchey et al. 2016). Our results showed that even when eye movements are forbidden and tonically suppressed, the oculomotor system could also be involved in manual IOR.

In the 200 ms gap condition, the fixation offset not only causes activation of the fixation system, but also acts as a kind of warning signal for an imminent target and provides enough time to prepare a response (Rolfs and Vitu 2007). Therefore, any conclusions about the reason for the IOR decrease should only be taken as tentative. On the other hand, the 0 ms gap condition, in which the fixation point disappears during the target presentation, is devoid of a warning signal; one might further argue that, in the 0 ms gap condition, participants could react rather to the offset of the fixation than to the onset of the target. However, in our experiments, responding participants could not rely on the disappearance of the fixation point because, contrary to the target, it was unpredictable (randomly during the task in 50% of trials). To maximize the chance to respond to a target, it is generally recommended to use catch trials, i.e., trials in which a target does not appear (Chica et al. 2014). A low proportion of catch trials could improve effect size by further reducing the probability of a response to the fixation offset; however, in the specific case of a task with a fixation offset and a simple manual response, catch trials may not be accurate as they can sometimes reduce the IOR effect itself (Chica et al. 2014) and by this could also reduce the effect size of IOR attenuation caused by the fixation offset. In addition, catch trials change a task from a simple detection task to a kind of Go-No go task and thereby attenuate oculomotor priming effects (Belopolsky and Theeuwes 2009; Smith and Casteau 2019) or involve some endogenous functions (i.e., the ability to inhibit a response) that could additionally decrease the contribution of the gap effect in both manual and saccadic IOR. For these reasons, we rejected their use in our experiments.

In line with other studies (Bompas et al. 2017), manual reaction times in our study were generally slower than saccades. Saccade initiation is faster than manual reactions, probably because both motor outputs rely on partially distinct dynamics on a cortical level (Filimon 2010; Buschman and Miller 2007) and these kinds of responses are also differently evoked by a peripheral stimulus. Saccades are more automatic and challenging to inhibit compared to manual responses (Malkinson and Bartolomeo 2018). One might argue that these response conditions could be equated by providing an additional stimulus that is presented simultaneously to the target in the opposite visual field. In this condition, an automatic saccade should not be triggered (Olk and Kingstone 2003; Lugli et al. 2016); however, this method, probably because the detection task was changed in the discrimination task, reduces the size of the IOR and involves more of an endogenous than exogenous attention (Kingstone and Pratt 1999).

It is important to note that many other studies have showed differences between saccadic and manual IOR (e.g. Hilchey et al. 2014, 2016; Hunt and Kingstone 2003; Kingstone and Pratt 1999; Taylor and Klein 2000; Zhang and Zhang 2011). However, a question arises as to whether this necessarily implies that manual IOR engages an attentional mechanism which is dissociated from sensory-motor functions. At least some of these findings could be explained by differences between the visual-motor processes engaged in both types of responses (Malkinson and Bartolomeo 2018). Recently, Smith and Casteau (2019) have also shown that some of the results that are interpreted as a dissociation between covert attention and oculomotor control could be alternatively explained by attenuation of the oculomotor priming effect that is caused using a high proportion of catch trials in the cuing task. Our results also indicate the limitations of the assumption that manual IOR is independent of saccadic processes.

Our results are consistent with the study of Souto and Kerzel (2009), who showed that target luminance impacts saccadic IOR in the same way as in manual IOR. As they showed the existence of a perceptual component in saccadic IOR and, therefore, needed to consider it as an attentional process, we provide evidence that manual IOR is related to eye preparation and should be considered a motoric process. Both studies showed that manual and saccadic IORs are more a part of the same kind of effector-based attention systems (Perry and Fallah 2017) that were postulated by the PToA (Rizzolatti et al. 1987, 1994) than they are part of two separate attention-based and motor-based IORs (Taylor and Klein 2000; Hilchey et al. 2016).

However, our results are in contrast to the study of Hunt and Kingstone (2003), who showed that the gap effect (0 ms gap) changes the saccade but not manual IOR. This could have been caused by the type of manual task used in both studies: we used a simple task, whereas Hunt and Kingstone used a choice keypress. The latter type of response is associated with various additional factors which could mask the gap effect. Deciding which keys to press is not mutually exclusive but requires additional resources to refrain from giving competitive responses (Bekkering et al. 1996). Choice keypress response could also cause some spatial compatibility effects, such as a faster response of the right hand compared to the left hand (Anzola et al. 1977). Besides, Hunt and Kingstone did not report any information about the participants’ hand dominance and this factor, if not controlled, could also additionally affect the results.

On the other hand, both sets of results may not be contradictory, but they require a further hypothesis according to which the choice and the simple types of response involve different visual selection processes. Due to the influence of endogenous factors, the choice response would be more related to non-motor attentional/perceptual processes, while the simple one would involve more basic, i.e., sensory-motor functions. However, this would mean the existence of two manual IORs: attention and motor. We do not know any concept that takes this type of distinction into account, but we consider it in terms of the distinction between exogenous and endogenous attention (Wright and Ward 2008). Casteau and Smith (2018) recently showed that—at least in some circumstances—endogenous attention, in contrast to exogenous, can be deployed independently of oculomotor control (see also: Smith and Schenk 2012). Some other authors also argue that, contrary to the previous findings (e.g., Posner and Cohen 1984), IOR could be evoked not only by exogenous but also by endogenous attention (e.g., Kingstone and Pratt 1999). In that context, it is possible that we observed exogenous attention, while Hunt and Kingston observed the endogenous type of manual IOR.

### The possible neuronal mechanism of IOR

Neurophysiological findings highlight the involvement of oculomotor structures in the IOR process. They mainly emphasize the role of the superior colliculus (Dorris et al. 1997; Munoz and Wurtz 1995; Bell et al. 2004; Dorris et al. 1999; Posner et al. 1985; Sereno et al. 2006). Hunt and Kingstone (2003) suggest that the gap effect decreases saccadic IOR via the intracollicular inhibition process (see also: Klein and Hilchey 2011). According to the attentional-based IOR hypothesis, these processes should not affect manual IOR, because the task required eye movements to be actively inhibited (e.g., Taylor and Klein 2000; Hilchey et al. 2016). However, even when eye movements are actively suppressed, visual selection could still depend on the preparation of eye movements that is generated by visual-motor neurons located in the intermediate layers of the SC (Rizzolatti et al. 1994; Craighero and Rizzolatti 2005; Ignashchenkova et al. 2004). Therefore, our results that show decreased manual IOR could be explained by the same collicular mechanism as in the case of saccadic IOR.

Although explaining IOR in terms of the intracollicular inhibition process is compelling, the IOR mechanism is more complicated and involves cortical areas (e.g., Dorris et al. 2002). Recently, Malkinson and Bartolomeo (2018), based on the extensive neurobiological literature, proposed a model (the so-called FORTIOR) explaining the cortical basis of IOR in a detection paradigm. According to this model, both saccadic and manual IOR arise mainly by activation of *the frontal eye field* (FEF) and *the inferior parietal sulcus* (IPS) circuit. When a cue is detected, its location is first registered in the priority map in the FEF. If a target does not appear after the limited temporal resolution of a single response (this equals the minimum SOA for IOR to occur; for a single saccade, it is 100–200 ms, and for single manual response, it is 200–300 ms), the previously activated location accumulates noise in the priority map of an IPS and reduces the signal-to-noise ratio (SNR). Adding noise in the previously activated location could filter out weak signals that appear later at the same location and deliver them to the other regions driven by the output of these maps, including response networks. This process would cause the IOR effect. It is important to note that, compared to the manual system, the saccadic system is less sensitive to noise; therefore, saccadic IOR is generally more reliable than manual IOR. However, this model does not assume the existence of separate attention processes for manual IOR; it states that both saccades and manual responses depend on the same IOR mechanism. This assumption is confirmed by our results relating to the interaction between the gap effect and both saccadic and manual IOR.

To summarize, we speculate that the gap effect could decrease in manual and saccadic IOR not only due to intracollicular inhibition, but also—at least partially—due to disruption of the priority map output in the FEF–IPS circuit.

### The interpretative limitations of the obtained results

The following limitations of our study should be considered. We measured manual IOR only in a simple response condition, and the hypothesis that choice response disturbs the gap effect and manual IOR interaction is only indirectly extrapolated from our results, and thus, it needs to be verified in further experiments. Second, our result does not falsify Hunt and Kingstone’s (2003) finding. It would require replication with a large sample size and detailed analysis of various factors that may affect the lack of gap and manual IOR interaction. Finally, even though our results show a limitation of the hypothesis according to which manual IOR is independent of the motoric component, some other studies show a dissociation between saccadic and manual IOR (e.g. Hilchey et al. 2014, 2016; Kingstone and Pratt 1999; Taylor and Klein 2000; Zhang and Zhang 2011). Therefore, it is still possible that, under certain conditions, attentional-based IOR, indeed, takes place.

## Conclusion

Our findings demonstrate that the gap effect reduces not only saccadic but also manual IOR; this could at least suggest that both share similar processes that are related to the preparation of eye movement and, therefore, stay in line with the PToA, which assumes that selective attention effects depend on eye movement preparation. Our results also contradict the hypothesis that assumes that eye movement restriction creates a condition for attentional processes which are independent of the motoric system. According to the state-of-the-art knowledge, it is still possible that, in some conditions, the IOR effect could also be produced by mechanisms which are not sensory-motor.

## Footnotes

1 For this experiment and for the second one to measure the IOR effect, we determined the sample size based on the meta-analysis of Chica et al. (2014). It shows that obtaining a strong (*d* Cohen > 0.8) IOR effect for manual condition requires at least *N* = 18.
